# Food insecurity and physical functioning in Boston area Puerto Rican older adults

**DOI:** 10.1017/S1368980022000301

**Published:** 2022-12

**Authors:** Lillian Kuehl, Jong Soo Lee, Deepika Dinesh, Muzi Na, Katherine L Tucker, Natalia Palacios

**Affiliations:** 1 Department of Public Health, Zuckerberg College of Health Sciences, University of Massachusetts Lowell, 61 Wilder Street, O’Leary Library, Suite 540-K, Lowell, MA 01854-3692, USA; 2 Center for Population Health, University of Massachusetts Lowell, Lowell, MA, USA; 3 Department of Mathematical Sciences, University of Massachusetts Lowell, Lowell, MA, USA; 4 Department of Nutritional Sciences, College of Health and Human Development, Pennsylvania State University, State College, PA, USA; 5 Department of Biomedical and Nutritional Sciences, University of Massachusetts Lowell, Lowell, MA, USA; 6 Department of Nutrition, Harvard School of Public Health, Boston, MA, USA; 7 Geriatric Research Education Clinical Center, Edith Nourse Rogers Memorial Veterans Hospital, Bedford, MA, USA

**Keywords:** Food insecurity, Functional limitations, Puerto Rican

## Abstract

**Objective:**

Prior studies have found evidence of a relationship between food insecurity and functional limitations among older populations in the USA.

**Design:**

This is a longitudinal investigation of food security in relation to functional limitations, assessed as Activities of Daily Living (ADL) and Instrumental Activities of Daily Living (IADL) scores.

**Setting:**

The Greater Boston, MA area.

**Participants:**

1461 Boston Puerto Rican Health study participants, predominantly (70·5 %) female and aged 57·1 years (sd ± 7·6) at baseline followed for 6·2 (sd ± 0·98) years.

**Results:**

In cross-sectional analysis at baseline, participants reporting severe food insecurity had greater functional limitations (higher ADL; *β* = 2·34; 95 % CI (1·48, 3·19)) and higher IADL (*β* = 1·17, 95 % CI (0·68, 1·65)) compared with food secure participants. In longitudinal linear mixed models, severely food insecure participants at baseline had greater functional limitations over 5 years, as assessed by ADL (*β* = 1·74; 95 % CI (0·95, 2·53); *P* < 0·001) and IADL (*β* = 0·93, 95 % CI (0·48, 1·38)) compared with food secure participants. However, baseline food security did not significantly alter the 5-year trajectory in ADL (*P*-interaction between baseline food security and time for ADL and IADL = 0·41 and 0·47, respectively).

**Conclusions:**

In this cohort of Boston area Puerto Rican adults, those who are food insecure had consistently higher ADL and IADL scores over time, compared with those who are food secure. Baseline food security did not appear to alter the trajectory in ADL or IADL score.

Food insecurity impacts approximately 10 % of the USA population and is prevalent in minority populations, including mainland Puerto Ricans^(1)^. Food insecurity has been linked to increased risk of obesity^(2)^, lower cognitive functioning^(3)^, major depression^(4)^ and other adverse outcomes^(5,6)^. Recent studies have linked food insecurity to functional limitations^(7–9)^ among older adults.

A healthy diet is essential for good health and functioning. As systematic review examining food insecurity and dietary quality in USA adults has shown that food insecure individuals consume fewer vegetables, fruits and dairy products compared with food secure individuals^(10)^. A recent study in older adults suggested that higher Mediterranean diet and Dietary Approaches to Stop Hypertension diet scores were associated with less disability, suggesting that quality diets may be effective at preventing functional disability in the older population^(11)^. A study of 1410 female participants reported that the adherence to the Mediterranean diet reduced the risk of incident disability by up to 50 %^(12)^. In another study, a plant-based dietary education intervention resulted in decreased pain and improvement in quality of life^(13)^. Intake of healthy foods, such as vegetables, was associated with better grip strength and frailty in a cohort study of 432 participants, while higher fruit juice intake was associated with worse grip strength and frailty^(14)^. A study of 14 260 elderly participants in Japan found that high intake of animal foods resulted in a higher risk of incident functional disability^(15)^. Food insecurity has also been associated with greater adiposity^(16)^, also a risk factor for functional limitations.

Reduction of ethnic and racial disparities in prevalence and incidence of chronic conditions is a top public health priority. Understanding of the relationship between food security and functional limitations in the elderly Puerto Rican population in the USA is limited, but necessary to improve the health status of this population. As the second largest Hispanic subgroup in the USA, Puerto Ricans have poorer health status and higher prevalence of several acute and chronic medical conditions, compared with non-Hispanic whites^(17)^. Within Hispanic subgroups, Puerto Ricans have been shown to be more likely to experience limitations in activities of daily living (ADL), compared with Cuban and Mexican Americans^(17)^. In Massachusetts, Puerto Rican elders have been shown to have significantly greater prevalence of chronic conditions such as type 2 diabetes, depression and physical disability than non-Hispanic whites^(17)^. In the Boston Puerto Rican Health Study cohort, food insecurity has previously been linked with worse cognitive functioning^(18)^.

Several studies have examined the relationship between food insecurity and functional limitations^(11–13,15)^, including in underserved populations in the USA^(14,16)^. However, to our knowledge, no previous investigation of food insecurity in relation to functional limitations has been conducted in Puerto Ricans, who represent a risk group of particular interest for this research because they are more likely to live in poverty and be food insecure than other Hispanics and Non-Hispanic Whites^(19)^. Thus, this study examined whether food insecurity was related to functional limitations, over 6·2 years (sd ± 0·98) of follow-up in a cohort of Puerto Rican adults living in the greater Boston, MA area.

## Methods

### Study population

This study examined prospectively whether food insecurity was related to functional limitations in a cohort of Puerto Rican adults living in the greater Boston, MA area. At baseline, participants were recruited from year 2000 Census blocks of high Hispanic density in the Boston, MA metropolitan area. Households with at least one adult between the ages of 45 and 75 years were contacted. One person per household was randomly selected to participate. More than 80 % of participants were selected in this manner, approximately 9 % were recruited through personal contact at major Puerto Rican events and the rest were found through media or personal referral. Participants unable to answer questions due to serious health conditions such as dementia were not included in the study. Study questionnaires were adapted from those used in the Third National Health and Nutrition Examination Survey (NHANES III)^(20,21)^, the Hispanic Health and Nutrition Examination Survey^(22,23)^ or the National Health Interview Survey Supplement on Aging^(24)^.

### Assessment of food security status

Food security was assessed using the US Department of Agriculture Food Security/Hunger scale^(25,26)^. Participants responded to 10 questions on food security over the previous 12 months, such as ‘(I/we) couldn’t afford to eat balanced meals’, ‘did you ever eat less than you felt you should because there wasn’t enough money to buy food?’ and ‘were you ever hungry but didn’t eat because you couldn’t afford enough food?’. Food security was categorised as a three-level ordinal variable: (1) food secure (answered >3 questions positively); (2) mild food insecurity (answered 3–5 positively) and (3) severe food insecurity (answered >6 questions positively).

### Assessment of functional limitations

Participants self-reported difficulty in performing daily activities, with modified Katz ADL (twelve questions) and Instrumental Activities of Daily Living (IADL, six questions) scales^(27)^. Questions focussed on the difficulty in performing tasks, such as the ability to get out of bed, get dressed, eat with cutlery and bathe or shower, ranging from 0 to 3, with 0 being no difficulty to 3 being impossible to do. These answers were summed to create scores that ranged from 0 to 36 for ADL or 0 to 18 for IADL, with higher scores representing greater disability.

### Covariates

Information on study covariates were collected at the study baseline as described previously^(28)^. During the interview, participants had their weight and height measured. BMI was calculated using weight (kg) divided by height (m) squared^(29)^. Frequency, history and type of alcohol consumption and smoking were assessed^(17)^. Participants provided fasting blood samples, drawn at home by a certified phlebotomist during the interview or soon thereafter. Diabetes status was defined as fasting plasma glucose ≥ 126 mg/dl or use of medication for diabetes^(30)^. Blood pressure was measured three times during the interview, and hypertension was defined as blood pressure ≥ 140/90 mmHg and/or a mean diastolic blood pressure ≥ 90 mmHg or use of blood pressure medication^(31)^. On the study questionnaire administered during the home interview, participants provided information on age and education level (<8^th^ grade, 9^th^–12^th^ grade, college/graduate school), physical activity and household income. Poverty status was calculated using poverty thresholds released yearly from the US Census Bureau^(17)^. Total annual household income was compared to the threshold based on the age of the head of household, participant’s family size and year of interview. The 120 % income to poverty ratio was derived by dividing the household income by the threshold. Smoking was categorised into three levels (current, former or never) in this analysis. A moderate drinking status was defined as having 1 drink/d for women, 1–2 drinks/d for men or daily alcohol intake estimated greater than 0 but less than 13·2 g for women, or daily alcohol intake estimated greater than 0 but less than 26·4 g for men; heavy drinking status was defined as exceeding these cut-offs. A physical activity score was calculated as the sum of hours spent on activities done in a typical 24-h day (heavy, moderate, light or sedentary activity and sleeping) multiplied by weighting factors that parallel the rate of oxygen consumption associated with each activity. Visit number (1/2/3) was modelled as a continuous variable in all linear mixed model analyses.

### Statistical analysis

Statistical analyses were performed in R version 3.6.0. Means and proportions, for all study variables, were computed by category of food security. Subsequently, general linear regression was used to examine the association between food security and ADL/IADL at baseline, adjusting for age, sex, education, BMI, presence of diabetes, smoking, alcohol use, physical activity score and income to poverty ratio. Additional sensitivity analyses, examining binary ADL and IADL (reports at least difficulty in any task *v*. no difficulty) as well as limitations in each individual ADL and IADL tasks were conducted using multivariable logistic regression.

Linear mixed effects models, with autoregressive covariance structure (due to the assumption of highest correlations between most closely adjacent times, as would be expected in this longitudinal study), with random intercepts and random slopes (function ‘lme’ in R) were used to examine the relation of food security change in ADL and IADL over three study visits spanning a mean of 6·2 years, as well as the individual components of these scores. Time was coded as a continuous variable in all models. All potential confounders were treated as fixed effects, and baseline measures were used.

To examine whether ADL or IADL, or their component score trajectory varied by baseline food security, a multiplicative term between visit number and food security level was added to the covariate-adjusted Linear mixed effects model for ADL and IADL. The *β* coefficient for this interaction term describes the effect of food security on change in ADL or IADL over time.

## Results

Food secure participants were older (mean = 57·3 years, sd = 7·6; *P* = 0·036), were less likely to be current smokers (*P* < 0·001), more likely to be former smokers (32·2 %, *P* < 0·001), more likely to have hypertension (70·5 %, *P* = 0·002), and more likely to have a college-level education (17·0 %, *P* = 0·032) and less likely to live at below 120 % income to poverty ratio, compared with those who were in the moderately and severely food insecure groups (Table 1).

**Table 1:** Baseline characteristics of 1461 Puerto Rican adults in Massachusetts, by food security status

	Baseline food security levels among covariates					
	Food secure 1065		Mild food insecurity 264		Severe food insecurity 132	
	*n*	%	*n*	%	*n*	%
ADL*						
Mean	3·46		5·52		5·59	
SD	4·84		5·59		5·25	
IADL†						
Mean	1·52		2·49		2·61	
SD	2·65		3·27		3·06	
Age (years)						
Mean	57·4		56·4		55·9	
SD	7·63		7·52		7·56	
Female	740	69·5 %	201	76·1 %	89	67·4 %
BMI‡ (kg/m^2^)						
Mean	31·8		32·6		30·8	
SD	6·52		6·91		7·02	
Income to poverty ratio§						
< 120 %	567	53·3 %	190	72·5 %	98	74·2 %
≥ 120 %	496	46·7 %	72	27·5 %	34	25·8 %
Education						
≤ 8th grade	484	45·5 %	127	48·1 %	67	50·8 %
9th–12th grade	398	37·4 %	105	39·8 %	55	41·7 %
Some college	181	17·03 %	32	12·1 %	10	7·58 %
Smoking						
Past	342	32·2 %	70	26·5 %	29	21·9 %
Current	226	21·3 %	79	29·9 %	51	38·6 %
Drinking‖						
Moderate	403	38·4 %	82	31·4 %	45	34·4 %
Heavy	81	7·71 %	15	5·75 %	19	14·5 %
PA score¶						
Mean	31·7		31·3		31·1	
SD	4·81		4·73		4·21	
Diabetes	420	40·2 %	104	40·8 %	43	34·1 %
Hypertension**	746	70·5 %	161	62·9 %	76	57·6 %

Values are presented as means (sd) for continuous variables, number in category (percentage) for categorical variables and median or dates (date of PD diagnosis and first symptoms).

* ADL summary score, sum of twelve questions.

† IADL summary score, sum of six questions.

‡ BMI = average of weight measurements divided by the square of the average of height measurements.

§ Poverty status was calculated using poverty thresholds released yearly from the US Census Bureau^(17)^. Total annual household income was compared to the threshold based on the age of the head of household, participant’s family size and year of interview. The 120 % income to poverty ratio was derived by dividing the household income by the threshold.

‖ Moderate drinking status = up to 1 drink/d for women, up to 2 drinks/d for men; heavy drinking is more than this.

¶ Physical activity score was derived from weighted time spent in sleeping and in vigorous, moderate and light activity.

** Hypertension = 1 for systolic blood pressure > 140, diastolic blood pressure > 90 or taking hypertension medication.

**Fig. 1:**
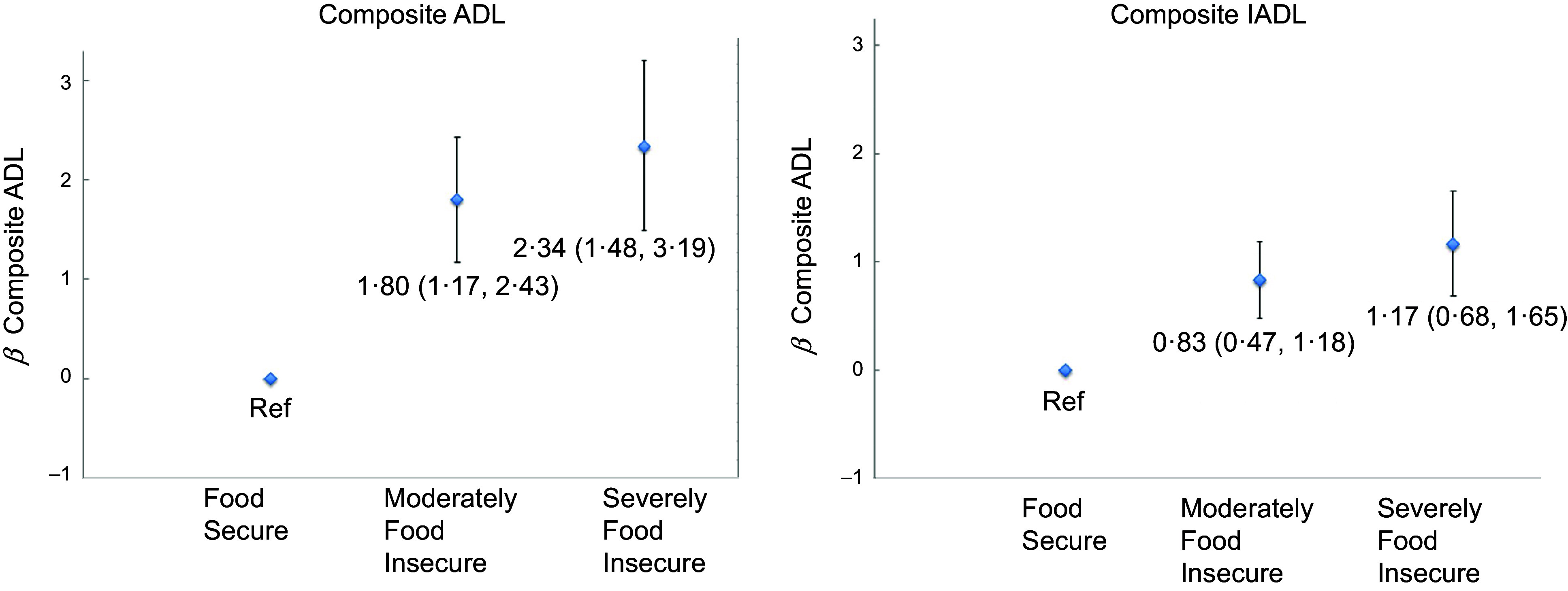
Cross-sectional association, at baseline, between food insecurity and ADL/IADL scores in 1461 Puerto Rican Adults Living in Massachusetts. Estimates adjusted for age, sex, education, BMI, smoking, alcohol frequency, physical activity score, diabetes and income to poverty ratio (120 %). *n* 118 observations deleted due to missingness composite ADL and IADL

At baseline, after adjusting for age, sex, education, BMI, 120 % income to poverty ratio status, smoking, alcohol intake, physical activity score and presence of diabetes, participants moderately food insecure (*β* = 1·80; 95 % CI (1·17, 2·43)) or severely food insecure (*β* = 2·34; 95 % CI (1·48, 3·19)) had higher ADL score, compared with those food secure (Fig. 1). Likewise, those moderately (*β* = 0·83; 95 % CI (0·47, 1·18)) or severely food insecure (*β* = 1·17; 95 % CI (0·68, 1·65)) had higher IADL scores than food secure participants (Fig. 1). Associations between food security and individual ADL and IADL components are shown in Supplemental Table 1.

In linear mixed models, treating subject as a random effect, severe (*β* = 1·74; 95 % CI (0·95, 2·53)) and moderate (*β* = 1·68; 95 % CI (1·10, 2·27)) food insecurity was associated with higher ADL scores, relative to food security. Likewise, severe (*β* = 0·93; 95 % CI (0·48, 1·38)) and moderate (*β* = 0·72; 95 % CI (0·39, 1·05)) food insecurity was associated with higher IADL scores, relative to those food secure, in linear mixed model analyses over 6·2 years follow-up (sd ± 0·98) (Table 2).

**Table 2:** Longitudinal associations between baseline food security and ADL and IADL

	Food secure	Moderate food insecurity		Severe food insecurity	
		*β*	95 % CI	*β*	95 % CI
ADL components					
Composite ADL*	Ref.	1·68	1·10, 2·27	1·74	0·95, 2·53
Composite ADL*visit†	Ref.	0·029	-0·17, 0·23	−0·15	-0·41, 0·11
IADL components					
Composite IADL*	Ref.	0·72	0·389, 1·05	0·93	0·48, 1·38
Composite IADL × visit‡	Ref.	−0·0063	−0·12, 0·11	−0·064	-0·22, 0·087

ADL, Activities of Daily Living; IADL,Instrumental Activities of Daily Living.

* Model 1: adjusted for age, visit, sex, education, BMI, smoking, alcohol frequency, physical activity score, diabetes (definition), and income to poverty ratio (120 %).

† Model additionally includes visit × ADL interaction term.

‡ Model additionally includes visit × IADL interaction term.

While food insecure participants had consistently higher levels of disability on both the ADL and IADL scales over time, this study did not find significant differences in ADL or IADL trajectories over time for severe food insecurity (*P*-interaction between ADL and time = 0·10; and between IADL and time = 0·09), and for moderate food insecurity (*P*-interaction between ADL and time = 0·21; and between IADL and time = 0·10) by baseline food security status.

## Discussion

In this study, baseline food insecurity was associated with greater disability, as assessed by ADL and IADL, both cross-sectionally and over 5 years, and after adjustment for age, sex, smoking, alcohol use, physical activity, education, BMI, 120 % income to poverty ratio status and diabetes. Although severely and moderately food insecure participants had higher ADL and IADL scores over time compared with food secure participants, baseline food insecurity did not appear to alter 5-year trajectories of ADL or IADL.

Prior studies of food insecurity, diet quality and functional limitations have been conducted among populations in the USA, Europe and Asia. Most prior studies have focussed on diet quality, and few have examined the relationship between food insecurity and functional limitations directly. Better adherence to the MIND (high in green leafy vegetables and berries), Mediterranean and Dietary Approaches to Stop Hypertension diets was associated with less ADL and IADL disability in a prospective 5-year study of 809 participants without functional limitations at baseline^(11)^. In the Bordeux, three-cities study of 1400 individuals, better adherence to the Mediterranean was associated with less incident disability (ADL) in women, but no effect was observed in men^(12)^. In the Ohasaki cohort study, high adherence to the Japanese diet (high in green and yellow vegetables, fish, green tea, rice; low in beef and pork) was associated with lower functional disability. Seifert *et al.*, examined food insufficiency directly and found that, among a cohort of African American and white welfare recipients, household food insufficiency was associated with worse physical health^(4)^. Among studies focussed on disadvantaged or minority cohorts, in population-based sample of middle-aged African-Americans, vegetable intake was associated with better performance on some aspects of ADL, such as grip strength and frailty^(14)^. To our knowledge, ours is the first study to focus exclusively on the Puerto Rican population. Ours is also one of few studies explicitly linking food insecurity, rather than a specific dietary pattern to functional limitation. Food insecurity is closely linked to poverty, which has been associated with higher likelihood of functional limitations^(32)^. Puerto Ricans are a socioeconomically vulnerable population: in 2010, they were estimated to have roughly 31 % lower income than the US median: approximately $36 000 among Puerto Ricans, compared with $50 000, and to have a higher proportion of families living below the poverty line, at 24 % in 2010^(19)^. Study participants were Boston area older Puerto Ricans, with a uniquely high burden of both poverty and food insecurity. Overall, 27·1 % of this sample were severely or moderately food insecure, 59·1 % lived at or below the 120 % income to poverty threshold at baseline and 19·8 % reported both food insecurity and poverty. Furthermore, this population had a particularly high burden of chronic disease, with 55·9 % obese (BMI > 30), 39·9 % with diabetes and 57·3 % exhibiting high depressive symptomatology (Center for Epidemiological Studies Depression (CESD) score > 16). Therefore, this population constitutes a uniquely high-risk group for potential adverse health effects of food insecurity on health, including ADL and IADL. In this study, poverty was adjusted for in statistical analyses using the poverty-to-income ratio. However, residual confounding by poverty is still a possibility.

In our study, food insecure individuals consistently experienced more functional disability, but greater baseline food insecurity did not appear to accelerate the disability trajectory over time. There has been limited research on the directionality of the relationship between food insecurity and functional limitations^(2)^. A report that relied on data from the National Health and Nutrition Survey reported that adults with disabilities were less likely to meet the 2010 Dietary Guidelines for Americans recommended intakes for saturated fat, fibre, vitamin A, vitamin C, Ca and K, compared with those without disability^(33)^. Of the numerous disability categories considered in the above study, including ADL, IADL, leisure and social activities, lower extremity mobility and general physical activities; of these, ADL were the disability category most strongly linked to an inability to meet the dietary guidelines. However, another study, using path analysis, reported that poor physical functioning was, in turn, associated with moderate and severe food insecurity in homes not enrolled in a food aid program or pension plan, suggesting a bidirectionality of the relationship between food insecurity and functional disability^(5)^. More work is needed to better understand the directionality of the relationship between food security and disability.

Strengths of the current study include three follow-up waves of longitudinal data on ADL and IADL scores, extensive covariate data and the unique study population of older Puerto Rican adults. The study benefited from detailed characterisation of food security status. The study also has several limitations. There was a substantial loss to follow-up, with over a third of the cohort lost to follow-up during the study period. Furthermore, even though poverty-to-income ratio was adjusted for in all analyses, residual confounding remains a possibility. While this study benefited for extensive information on covariates, allowing for adjustment for potential confounders, there is still a possibility of residual confounding. Finally, due to the unique population in this study, generalisability may be limited, and similar analyses should be conducted in other populations.

In summary, in this cohort of Boston Area Puerto Ricans, food insecure participants had consistently higher ADL and IADL scores over time, compared with food secure participants. However, baseline food security did not appear to have an effect on the 5-year trajectory in ADL or IADL scores. To our knowledge, this is the first study that has examined the relationship between food insecurity and functional limitations in Puerto Ricans. Additional research on the directionality of the relationship between food security and functional limitations is necessary.

## Supplementary material

For supplementary material accompanying this paper visit https://doi.org/10.1017/S1368980022000301. click here to view supplementary material
