# Supplementary material for: Food insecurity and physical functioning in Boston area Puerto Rican older adults
Source: Public Health Nutr. 2022 Aug 5;25(12):3520–6. doi: 10.1017/S1368980022000301 (PMC9991585; doi:10.1017/S1368980022000301)
Supplement: Supplementary file 1 [file S1368980022000301sup001.docx]

**Supplemental Table 1. Cross-sectional association, at baseline, between food insecurity and ADL/IADL Binary Score and Individual Components in 1461 Puerto Rican Adults Living in Massachusetts.**

|  | **Food Secure** | **Moderate Food Insecurity** | | **Severe Food Insecurity** | |
| --- | --- | --- | --- | --- | --- |
|  |  | Odds Ratio | 95% CI | Odds Ratio | 95% CI |
| ADL Overall^1^ | Ref. | 2.04 | 1.39, 2.97 | 2.66 | 1.57, 4.49 |
| ADL1^2^ | Ref. | 1.74 | 1.27, 2.38 | 1.66 | 1.09, 2.54 |
| ADL2^2^ | Ref. | 1.59 | 1.13, 2.22 | 2.32 | 1.44, 3.74 |
| ADL3^2^ | Ref. | 2.17 | 1.53 3.09 | 2.64 | 1.66, 4.20 |
| ADL4^2^ | Ref. | 1.72 | 1.11, 2.66 | 1.77 | 0.99, 3.25 |
| ADL5^2^ | Ref. | 1.91 | 1.41, 2.59 | 1.92 | 1.27, 2.89 |
| ADL6^2^ | Ref. | 1.35 | 0.81, 2.25 | 1.94 | 1.04, 3.64 |
| ADL7^2^ | Ref. | 1.74 | 1.23, 2.46 | 2.47 | 1.57, 3.88 |
| ADL8^2^ | Ref. | 2.09 | 1.41, 3.11 | 3.02 | 1.83, 4.98 |
| ADL9^2^ | Ref. | 1.74 | 1.08, 2.81 | 1.37 | 0.69, 2.72 |
| ADL10^2^ | Ref. | 2.12 | 1.52, 2.97 | 3.53 | 2.29, 5.45 |
| ADL11^2^ | Ref. | 2.35 | 1.69, 3.26 | 2.48 | 1.60, 3.83 |
| ADL12^2^ | Ref. | 1.86 | 1.31, 2.66 | 2.71 | 1.72, 4.27 |
|  |  |  |  |  |  |
| IADL Overall^1^ | Ref. | 2.03 | 1.49, 2.76 | 2.31 | 1.52, 3.49 |
| IADL1^2^ | Ref. | 1.97 | 1.45, 2.67 | 2.56 | 1.69, 3.88 |
| IADL2^2^ | Ref. | 2.42 | 1.65, 3.56 | 3.306 | 2.03, 5.39 |
| IADL3^2^ | Ref. | 1.86 | 1.27, 2.73 | 2.79 | 1.74, 4.46 |
| IADL4^2^ | Ref. | 1.87 | 1.35, 2.59 | 2.09 | 1.35, 3.22 |
| IADL5^2^ | Ref. | 1.53 | 1.11, 2.10 | 2.16 | 1.42, 3.28 |
| IADL6^2^ | Ref. | 1.28 | 0.69, 2.39 | 2.14 | 1.00, 4.55 |

Logistic regression models adjusted for age, sex, education (levels), BMI, smoking (levels), alcohol frequency (levels), physical activity score (levels), diabetes and income to poverty ratio (120%)

^1^ Summary ADL or IADL score, estimating odds of some or considerable impairment vs. no impairment (eg. sum of ADL components = 0 vs. 1 or more)

^2^Individual ADL or IADL components, estimating odds of any vs. no difficulty on a given task:

ADL1 Walking for a quarter of a mile (2 - 3 blocks)?

ADL2 Walking up 10 steps without resting?

ADL3 Getting outside?

ADL4 Walking from one room to another on the same level?

ADL5 Getting out of bed or chairs?

ADL6 Eating, like holding a fork, cutting food or drinking from a glass?

ADL7 Dressing yourself, including tying shoes, working zippers and doing buttons?

ADL8 Bathing or showering?

ADL9 Using the toilet, including getting to the toilet?

ADL10 Using a manual can opener?

ADL11 Opening a frozen food package?

ADL12 Opening a milk carton or orange juice carton?

IADL1 Doing chores around the house (like vacuuming, sweeping, dusting, or straightening up)?

IADL2 Preparing your own meals?

IADL3 Managing your money (such as keeping track of your expenses or paying bills)?

IADL4 Shopping for personal items (such as toiletry items or medications)?

IADL5 Food Shopping?

IADL6 Using the telephone?
